# Contribution of PGC-1α to Obesity- and Caloric Restriction-Related Physiological Changes in White Adipose Tissue

**DOI:** 10.3390/ijms22116025

**Published:** 2021-06-02

**Authors:** Masaki Kobayashi, Yusuke Deguchi, Yuka Nozaki, Yoshikazu Higami

**Affiliations:** 1 Laboratory of Molecular Pathology and Metabolic Disease, Faculty of Pharmaceutical Sciences, Tokyo University of Science, 2641 Yamazaki, Noda 278-8510, Japan; 3B20547@ed.tus.ac.jp (Y.D.); nozaki@rs.tus.ac.jp (Y.N.); 2 Research Institute for Biomedical Sciences, Tokyo University of Science, 2669 Yamazaki, Noda 278-8510, Japan

**Keywords:** PGC-1α, obesity, caloric restriction, white adipose tissue

## Abstract

Peroxisome proliferator-activated receptor γ coactivator-1 α (PGC-1α) regulates mitochondrial DNA replication and mitochondrial gene expression by interacting with several transcription factors. White adipose tissue (WAT) mainly comprises adipocytes that store triglycerides as an energy resource and secrete adipokines. The characteristics of WAT vary in response to systemic and chronic metabolic alterations, including obesity or caloric restriction. Despite a small amount of mitochondria in white adipocytes, accumulated evidence suggests that mitochondria are strongly related to adipocyte-specific functions, such as adipogenesis and lipogenesis, as well as oxidative metabolism for energy supply. Therefore, PGC-1α is expected to play an important role in WAT. In this review, we provide an overview of the involvement of mitochondria and PGC-1α with obesity- and caloric restriction-related physiological changes in adipocytes and WAT.

## 1. Introduction

Peroxisome proliferator-activated receptor γ (PPARγ) coactivator-1 α (PGC-1α) is a master transcriptional cofactor for mitochondrial biogenesis. PGC-1α was discovered as a PPARγ-interacting protein that is expressed preferentially in brown adipose tissue (BAT) [1,2]. PGC-1α binds to transcription factors, such as nuclear respiratory factor (NRF)-1, NRF-2, and estrogen-related receptor α (ERRα), thereby coactivating downstream genes [3,4,5,6]. NRF-1 and NRF-2 transcriptionally regulate various mitochondrial genes involved in the respiratory chain, and replication and transcription of mitochondrial DNA (mtDNA), which encodes part of proteins comprising respiratory chain complexes [5]. ERRα modulates β-oxidation and the tricarboxylic acid cycle, as well as mitochondrial biogenesis [6,7,8]. Among the downstream mediators of PGC-1α, transcription factor A mitochondria (TFAM) is a major factor responsible for mitochondrial biogenesis [9]. TFAM coats and stabilizes individual mtDNA molecules and also binds to a specific site of mtDNA, which in turn induces promoter activity during initiation of transcription [10]. Therefore, TFAM is required for replication and transcription of mtDNA. In addition to mitochondrial biogenesis, PGC-1α is involved in responses to oxidative stress via induction of sirtuin-3 (SIRT3), which is a member of the SIRT family. SIRT3 is a deacetylase that localizes within the mitochondrial matrix and plays a pivotal role in β-oxidation and antioxidative reactions by modulating acetylation levels of mitochondrial enzymes (e.g., long-chain acyl coenzyme A dehydrogenase and manganese superoxide dismutase) [11,12]. PGC-1α activates transcription of the *Sirt3* gene through binding of ERRα to the *Sirt3* proximal promoter [13]. These findings suggest that PGC-1α contributes to not only mitochondrial biogenesis, but also to metabolic pathways and oxidative stress responses.

Obesity, which is characterized by excess body weight or fat mass due to over-nutrition, causes disturbances in the metabolic, endocrine, and immune systems in the whole body. These obesity-induced abnormalities pose serious health problems, including type 2 diabetes mellitus (T2DM), non-alcoholic fatty liver disease, and cardio- and cerebrovascular diseases [14,15,16]. Obesity induces many cellular stresses and inflammatory signaling pathways by excess or ectopic accumulation of fat in various tissues, resulting in insulin resistance and hepatic steatosis [16,17]. Moreover, emerging evidence has indicated a relationship of mitochondrial dysfunction with oxidative stress and systemic inflammation in the obese condition [18].

Caloric restriction (CR) is a reproducible and simple experimental manipulation that delays onset of numerous age-associated pathophysiological changes and prolongs the median and maximum lifespan in various laboratory models (e.g., yeast, worms, and mammals) [19,20]. A recent report showed that CR exerted beneficial effects on non-human primates, which suggested the effectiveness of CR in humans [21]. Therefore, CR mimetics, which mimic the underlying mechanisms of the beneficial effects of CR, have attracted attention [22]. Previous studies have described that beneficial effects of CR including various physiological and molecular mechanisms [23]. These mechanisms include enhancement of mitochondrial biogenesis, suppression of inflammation, mitigation of oxidative stress, suppression of growth hormone/insulin-like growth factor (GH/IGF-1) signaling, mechanistic target of rapamycin complex 1 activity, and activation of sirtuin. Of note, several of these mechanisms are directly or indirectly relevant to mitochondria. In fact, we have shown that mitochondrial regulation in white adipose tissue (WAT) contributes to the beneficial effects of CR [24,25].

WAT largely comprises adipocytes, but also comprises adipose-derived stem cells (ADSC), fibroblasts, macrophages, and other immune cells [26]. Adipocytes store excess energy in the form of triglycerides (TG). Adipocytes are endocrine cells that secrete adipokines, such as adiponectin, leptin, and pro-inflammatory cytokines [26,27]. Adiponectin is a representative beneficial adipokine with the ability to improve insulin resistance by activating AMP-activated protein kinase (AMPK) in skeletal muscle and the liver [26,28]. Leptin participates in diverse physiological processes, including energy homeostasis, reproduction, angiogenesis, and the immune system [29]. Pro-inflammatory adipokines, such as interleukin-6, tumor necrosis factor α (TNFα), serpin family E member 1, and monocyte chemoattractant protein-1, cause inflammatory reactions and insulin resistance [30,31,32,33]. The secretory profile and characteristics of WAT vary depending on the size of adipocytes. Hypertrophic adipocytes with a large amount of TG, which are observed in obese individuals, preferentially secrete pro-inflammatory adipokines, thereby inducing local inflammation and insulin resistance [34,35]. In contrast, small adipocytes with a modest amount of TG, which are frequently observed in CR models, secrete more adiponectin and less monocyte chemoattractant protein-1 and TNFα, leading to improved insulin sensitivity in the whole body [36]. The findings mentioned above suggest that obesity- or CR-associated differences in the characteristics of WAT greatly contribute to systemic metabolism.

As mentioned above, mitochondrial regulation is closely implicated in obesity-related pathology and the effects of CR, supporting the relationship between PGC-1α and metabolic states in WAT. Despite many reviews of PGC-1α, few papers currently focus on and comprehensively highlight its link with metabolic states. Therefore, to provide novel insights into the physiological significance of PGC-1α, this review outlines the functions of mitochondria and the involvement of PGC-1α with obesity- and CR-related physiological changes in WAT, which is a tissue involved in whole-body metabolism.

## 2. Mitochondria and PGC-1α in WAT during Obesity or CR

### 2.1. Overview of Mitochondrial Roles in WAT

Adipocytes in WAT contain small and elongated mitochondria in the narrow cytoplasmic space, resulting from a large, unilocular lipid droplet formed by TG [37,38]. White adipocytes have been typically suspected to have a small number of mitochondria [37]. Some previous reports and reviews have described the relationship between mitochondria and white adipocyte-specific functions, such as adipocyte differentiation (or adipogenesis), lipid homeostasis, and insulin sensitivity [39,40]. Wilson-Fritch and colleagues showed that in 3T3-L1 cells (mouse fibroblasts with the ability to differentiate into adipocytes under specific conditions), mitochondrial capacity is activated depending on differentiation of adipocytes [41]. This finding could be explained by the requirement of a large amount of energy for the differentiation process of adipocytes [41]. During differentiation of adipocytes, transcriptional factors, including CCAAT/enhancer binding proteins, ERRα, and PPARγ, are sequentially induced, which in turn promote maturation of adipocytes [42,43,44]. Interestingly, PGC-1α is also upregulated in fully differentiated 3T3-L1 adipocytes, indicating an association between adipogenesis and mitochondrial biogenesis [44]. This is supported by the finding that some mouse models with adipose tissue-specific deletion of mitochondria-related factors show lipoatrophy or lipodystrophy, which represent loss of WAT [45].

Mitochondria generally contribute to the regulation of lipid metabolism via β-oxidation as follows: Fatty acid (FA) is transported into mitochondria through carnitine palmitoyl transferases in the form of acyl-coenzyme A (acyl-CoA) [46]. In the mitochondrial matrix, acyl-CoAs are oxidized into acetyl-CoAs, which are eventually metabolized in the tricarboxylic acid (TCA) cycle [46]. Furthermore, mitochondria reportedly provide the key factors for de novo synthesis of both FA and TG, including citrate, glycerol 3-phosphate (G3P), and nicotinamide adenine dinucleotide phosphate (NADPH) [47,48]. In nutrient-rich conditions, citrate, which is an intermediate of the TCA cycle in mitochondria, is shuttled into the cytosol through the citrate carrier [49]. Citrate is then converted into oxaloacetate and acetyl-coenzyme A by ATP-citrate lyase [50]. Subsequently, acetyl-CoA is used for FA synthesis [50]. In fact, PGC-1α reportedly induces lipogenesis by the production of citrate in tumors, thereby promoting tumor growth [51]. In addition, oxaloacetate in the cytosol is converted into phosphoenolpyruvate (PEP) by PEP carboxykinase (PEPCK), resulting in G3P formation [52,53]. G3P is dephosphorylated into glycerol, which is esterified with FA to form TG [52]. In addition to citrate and oxaloacetate, malate is an intermediate of the TCA cycle, which is important for lipogenesis. Malate is transported into the cytosol and converted into pyruvate by malic enzyme [54]. In this process, NADPH, a coenzyme for FA synthesis, is generated [55]. These findings underscore that mitochondria regulate both lipid metabolism and lipogenesis.

Insulin sensitivity is closely involved in lipogenesis via transcriptional regulation of FA synthesis-related factors in white adipocytes [56,57]. The following studies are examples indicating the association between mitochondria and insulin sensitivity. Wang and colleagues showed that inhibitors of mitochondrial respiratory complexes or knockdown of *Tfam* attenuate insulin signaling in WAT [58]. However, primary adipocytes established from subcutaneous WAT of obese patients have been shown to exhibit increased mitochondrial respiration despite their insulin resistance [59]. The authors described that this increase is a compensatory reaction for attenuated glucose metabolism due to insulin resistance [59]. Therefore, although it remains to be determined whether mitochondria are directly or indirectly associated with insulin sensitivity, mitochondria may participate in lipogenesis via the regulation of insulin signaling in addition to the supply of metabolic intermediates.

### 2.2. Function and Regulation of PGC-1α in Obese WAT

Obesity impairs mitochondrial biogenesis and oxidative metabolism in WAT [41]. White adipocytes isolated from ob/ob mice or db/db mice (genetic obesity models) show a comprehensive decrease in the expression of genes encoding mitochondrial proteins and a decline in oxygen consumption and citrate synthase activity [42,60,61]. In agreement with the results of experimental animal models, several studies have provided evidence that WAT in humans with obesity shows low levels of mtDNA and proteins comprising mitochondrial respiratory complexes [41,62,63,64]. A study showed that PGC-1α was decreased in WAT of obese rodents, and *Nrf1* and *Tfam* mRNA expression, protein abundance of cytochrome c oxidase subunit IV, mtDNA levels, and mitochondrial density were also downregulated [65]. This study also identified TNFα as a cause of obesity-induced decrease in PGC-1α expression and mitochondrial dysfunction [65]. Similarly, human studies have shown that obesity attenuates PGC-1α expression in WAT [63,64,66,67]. Furthermore, Kleiner and colleagues found that adipose tissue-specific *Pgc-1α* knockout (KO) mice fed a high-fat diet displayed decreased levels of genes involved in oxidative phosphorylation and β-oxidation, impaired glucose tolerance, and insulin resistance [68]. These findings suggest that downregulation of PGC-1α in WAT is associated with obesity-related disturbance of whole-body metabolism, although PGC-1α is endogenously expressed at low levels in WAT [2].

Adipocytes observed in obese WAT generally show an increased cell size (hypertrophy), rather than cell number (hyperplasia) [41]. As described above, hypertrophic adipocytes trigger insulin resistance. Conversely, hyperplasia of WAT represents the presence of many small adipocytes, which is associated with improvement of insulin sensitivity [69]. Hyperplasia is associated with the induction of adipocyte differentiation, namely adipogenesis [41]. Because of the relationship between adipogenesis and mitochondrial biogenesis, mitochondrial regulation by PGC-1α is predicted to be involved in the pathology of obesity via morphological changes in white adipocytes. However, markers of white adipocyte differentiation remain unchanged in WAT of adipose tissue-specific *Pgc-1α* KO mice [68]. Hypertrophy and hyperplasia are known to be regulated by a complicated paracrine mechanism between ADSCs and mature adipocytes [70]. Additionally, to the best of our knowledge, no study has shown direct participation of PGC-1α in white adipocyte differentiation. PGC-1α is widely accepted as a marker of transdifferentiation of white into brown adipocytes (known as “beiging”) [71]. Therefore, mitochondrial biogenesis regulated by PGC-1α may play an important, but not necessary, role in the differentiation of white adipocytes.

Negative regulators of PGC-1α, including receptor-interacting protein 140 (RIP140), p53, DNA methyltransferase 3 (DNMT3), and MYB binding protein (p160) 1a (MYBBP1a) and Parkin interacting substrate (PARIS), are considered to be responsible for obesity-induced downregulation of PGC-1α [72]. Hence, we explain the relationship between each negative regulator and PGC-1α in the following paragraphs.

RIP140 is a coregulator of a number of nuclear receptors and several other transcription factors in various tissues and organs [73,74]. RIP140 interacts with PGC-1α and negatively regulates its transcriptional activity [74]. Leonardsson and colleagues showed that *Rip140* expression was increased in relation to differentiation into adipocytes in 3T3-L1 cells [75]. Subsequently, Powelka and colleagues reported that RIP140 suppressed oxidative metabolism and mitochondrial biogenesis in adipocytes [76]. Moreover, RIP140 depletion has been shown to prevent obesity-induced glucose intolerance and insulin resistance in mice [75,76]. These findings indicate the substantial contribution of RIP140 to the pathology of obesity via downregulated PGC-1α in WAT.

The tumor suppressor p53 is responsive to various stresses, and accumulated evidence has also strongly suggested that p53 greatly contributes to mitochondrial regulation [77,78]. Our laboratory has identified a suppressive effect of p53 on PGC-1α expression levels and mtDNA content in 3T3-L1 adipocytes, but not in C2C12 myocytes [79]. Additionally, Maser and colleagues found that telomere dysfunction-induced p53 repressed PGC-1α expression, which led to mitochondrial dysfunction [80]. Several p53-deficient mouse models have been reported to be resistant to obesity and show upregulated PGC-1α expression. Fat-specific *p53* KO mice show improved insulin sensitivity by preventing senescence-like features in WAT [81]. A research group described that PGC-1α and mitochondrial genes were increased in subcutaneous WAT of systemic *p53* KO mice, which showed low WAT weight and improved glucose tolerance [82]. Similarly, in vivo transient repression of p53 by the CreERT2/loxP system or administration of pifithrin-α (inhibitor of p53 transcriptional activity) induce PGC-1α expression in WAT, and reduce the respiratory exchange ratio [83].

DNMT3 plays a central role in epigenetic modifications of the genome via DNA methylation [84]. Barrès and colleagues showed that, in skeletal muscle from patients with T2DM, DNMT3-related non-CpG hypermethylation occurred in the *PGC-1α* promoter, which resulted in decreased PGC-1α gene expression and mtDNA levels [85]. Furthermore, DNMTs are increased in WAT in patients with obesity, thereby inducing the methylation rate of the promoter region of the Krüppel-like factor 4 (*KLF4*) gene (an anti-inflammatory factor) and suppressing this expression [86]. These studies suggest that DNMT3 is associated with obesity-related downregulation of PGC-1α expression and mitochondrial function in adipocytes or WAT, despite no direct evidence. MYBBP1a represses transcriptional activity of PGC-1α by direct interaction in skeletal muscle [87]. PARIS, which is a transcriptional factor known as zinc-finger protein 746, attenuates *PGC-1α* expression by binding to insulin responsive sequences in the *PGC-1α* promoter region [88]. Whether MYBBP1a and PARIS are expressed in adipocytes or WAT remains to be evaluated. However, these reports raise the possibility of the contribution of MYBBP1a or PARIS to a decrease in PGC-1α in obese WAT.

### 2.3. Function and Regulation of PGC-1α in WAT during CR

Studies that have analyzed involvement of CR with mitochondria or PGC-1α in WAT are currently limited. In fact, many studies have addressed the effects of CR on mitochondrial biogenesis and efficiency in skeletal muscle, brain, or BAT in rodent models [89]. However, our proteome analysis showed that CR enhanced mitochondrial biogenesis in WAT, but not in BAT [90]. In agreement with our result, a microarray study performed by Linford and colleagues showed CR-induced upregulation of genes involved in mitochondrial oxidative phosphorylation in WAT [91]. Nisoli and colleagues reported that CR increased *Pgc-1α* and mtDNA levels in WAT [92]. Similarly, Pardo and colleagues showed that CR-induced upregulation of mitochondrial genes in WAT depended on PGC-1α and PGC-1β using double-KO mice [93]. Another study also showed that CR was more likely to upregulate mRNA levels of thermogenic genes and *Pgc-1α* in WAT than in BAT, which represented induction of beiging [94]. These studies suggest the importance of mitochondrial regulation and PGC-1α in WAT for the effects of CR.

The suppression of negative regulators of PGC-1α is likely to be a mechanism involved in CR-upregulated PGC-1α expression. For example, CR reduced TNFα gene expression levels in WAT [95]. Likewise, transcriptome analysis of WAT from CR mice showed p53 gene expression was suppressed by CR [96]. Additionally, Wang and colleagues demonstrated that aging induced RIP140 expression, while CR prevented this induction in WAT [97]. The authors also revealed that *Rip140* KO female mice exhibit an extended lifespan [97]. These studies support the proposal that the suppression of negative regulators can contribute to CR-upregulated PGC-1α expression and mitochondrial biogenesis in WAT.

Among the positive regulators of PGC-1α, SIRT1 and AMPK are representative CR-related mediators [98,99]. AMPK is activated in response to an energy expenditure-induced increase in the AMP/ATP ratio, thereby assisting in catabolic processes to supply energy [100]. AMPK enhances the transcriptional activity of PGC-1α by promoting its phosphorylation, and also upregulates *Pgc-1α*, resulting in activation of mitochondrial metabolism and biogenesis [101,102]. SIRT1 is an NAD-dependent deacetylase that greatly contributes to the beneficial effects of CR [103,104]. SIRT1 is involved in mitochondrial biogenesis by stimulating activity of PGC-1α via its deacetylation, despite the controversy over whether this involvement is critical for regulation of PGC-1α [105,106,107,108]. SIRT1-induced deacetylation of PGC-1α is also regulated by AMPK [109]. The AMPK/SIRT1/PGC-1α axis plays a central role in CR-related regulation of mitochondrial biogenesis in various organs and tissues, and is accepted as a main target of CR mimetics [22]. Resveratrol, which is a natural polyphenol with the ability to activate SIRT1, exerts beneficial effects on metabolic disorders as shown by some clinical trials [110]. Notably, resveratrol fails to increase mitochondrial gene levels in skeletal muscle of muscle-specific *Pgc-1α* KO mice, but there are systemic effects of this compound on energy expenditure [111]. Resveratrol also upregulates *Pgc-1α* expression and lipid metabolism-related genes in WAT [111]. Moreover, several reviews have described that natural polyphenols induce beiging of WAT [112,113,114]. Taken together, these studies suggest that activation of the AMPK/SIRT1/PGC-1α axis in WAT is likely to be involved in the systemic metabolic effects of CR.

In addition to the AMPK/SIRT1/PGC-1α axis, we have recently identified sterol regulatory element-binding protein 1c (SREBP1c) and fibroblast growth factor 21 (FGF21) as WAT-specific mediators of CR-induced mitochondrial biogenesis [25,115]. Suppression of GH/IGF-1 signaling is a well-known and major mechanism of the beneficial effects of CR [116]. Although animal models with suppressed GH/IGF-1 signaling generally live longer, interestingly, CR can further extend longevity in these animals [117,118]. This finding indicates that GH/IGF-1 signaling-independent mechanisms are involved in the effects of CR [117,118]. Our microarray analysis of WAT in ad libitum-fed rats, CR rats, and dwarf rats with suppressed GH/IGF-1 signaling showed that CR induced expression of genes involved in FA synthesis and SREBP1c [119]. SREBP1c is a master transcriptional factor of these genes. To evaluate the contribution of SREBP1c to the effects of CR, we examined the phenotypes of *Srebp-1c* KO mice. In CR conditions, *Srebp-1c* KO mice showed loss of upregulation of genes involved in FA synthesis in WAT and suppression of extension of the lifespan [24]. In this study, we discovered that CR-induced upregulation of *Pgc-1α* and mtDNA levels were suppressed in WAT of *Srebp-1c* KO mice. Consistently, *Srebp-1c* KO mouse embryonic fibroblasts show decreased *Pgc-1α* and mtDNA levels, suggesting SREBP-1c-dependent regulation of *Pgc-1α* [24]. Furthermore, we found that SREBP-1c directly bound to the *Pgc-1α* promoter region, thereby activating its gene expression [24]. Our recent study also showed increased levels of PGC-1α in *Srebp-1c-*overexpressing 3T3-L1 adipocytes [120]. These findings suggest that SREBP-1c is a direct inducer of PGC-1α expression and mitochondrial biogenesis.

FGF21 is a member of the endocrine FGF superfamily of which expression is highest in the liver [121]. However, *Fgf21* is expressed in other tissues or organs, such as WAT, BAT, and skeletal muscle [122]. Circulating FGF21, which is mostly secreted from the liver, binds to FGF receptor (FGFR) and the beta-klotho (KLB) receptor complex in target tissues, which in turn regulates glucose and lipid metabolism [123]. Notably, FGF21 signaling induces *Pgc-1α* expression in adipocytes [124]. We recently reported that CR upregulated *Fgf21* and *Klb* expression, as well as glucose transporter 1 and *Pgc-1α* expression, which are downstream genes of FGF21 signaling, in rat WATs [115]. These genes were also increased in *Fgf21*-overexpressing adipocytes [115]. Subsequently, we showed a decrease in *Pgc-1α* levels in *Fgf21* KO mouse embryonic fibroblasts [120]. These results suggest that FGF21 contributes to CR-induced upregulation of *Pgc-1α* in WAT probably via an autocrine mechanism. Additionally, we found that *Fgf21* levels were upregulated in *Srebp-1c*-overexpressing adipocytes and downregulated in WAT of *Srebp-1c* KO mice [120]. This observation is consistent with a study of Véniant and colleagues who found that fat-specific SREBP-1c transgenic mice showed an increase of *Fgf21* in WAT [125]. Therefore, SREBP-1c likely increases PGC-1α expression not only directly, but indirectly, via FGF21.

## 3. Discussion

Accumulated evidence has shown functional changes of mitochondria in WAT in relation to the systemic metabolic state, including obesity and CR. The cause-and-effect relationship between PGC-1α and the influence of obesity or CR on WAT is complicated. We aimed to provide insight into the physiological significance of alterations in PGC-1α in WAT as follows.

Obesity is regarded as an over-nutrition-induced state. In the early phase of obesity, adipogenesis and lipid anabolism need to be extremely induced to metabolize excess nutrition, probably resulting in over-activation of mitochondria in WAT. The persistence of such a condition in mitochondria is generally considered to trigger production of more reactive oxygen species (ROS), which are byproducts of oxidative phosphorylation. In fact, several studies have shown that obesity upregulates mitochondrial ROS levels in WAT [126,127]. At low levels, ROS play an important role in insulin signal transduction and differentiation of adipocytes [128,129]. In contrast, higher ROS levels cause oxidative stress, mitochondrial dysfunction, and inflammation [130]. Therefore, mitochondria are damaged by being exposed to accumulated ROS depending on the progress of obese conditions. ROS also induce mutations in mtDNA [131]. Although PGC-1α is involved in not only mitochondrial biogenesis, but improvement of oxidative stress, obesity-induced downregulation of PGC-1α may represent a defensive reaction to accumulation of abnormal mtDNA that is likely to further exacerbate mitochondrial function.

CR is regarded as a chronic and mild energy shortage condition. CR significantly enhances lipid catabolism in the whole body, which allows more efficient systemic metabolism to compensate for an energy shortage [132]. A previous study and our study suggest that WAT contributes to this metabolic shift by promoting de novo synthesis of lipids, which are a more efficient source of energy than carbohydrates [25,132]. Considering that mitochondria play an important role in lipogenesis as mentioned above, CR-induced PGC-1α in WAT may represent enhanced de novo lipogenesis, rather than lipid catabolism by β-oxidation. This notion is supported by the fact that SREBP-1c, which is a master regulator of FA synthesis, positively regulates *Pgc-1α* expression in WAT [24,25].

At present, the above-mentioned topics remain to be fully clarified. In this manuscript, we review findings mainly based on studies that focus on the physiological changes from normal conditions to obese or CR conditions. However, there are many studies that demonstrate CR-related changes in obese conditions [133,134,135,136,137]. Investigation of the effects of CR on obesity physiology is important for evaluating the clinical significance of CR. Thus, further accumulation of relevant findings will aid in addressing the cause-and-effect relationship between PGC-1α and obesity or CR.

Methodological limitations of the current research mentioned in this review include phenotypic differences between systemic and conditional KO mice. It is conceivable that metabolic alterations are especially susceptible to these differences. For instance, fat-specific conditional KO mice may provide experimental models suitable for more accurate analysis of targeted genes. Currently, the general fat-specific KO mice are not WAT-specific deficient models, because adiponectin-Cre, the transgene usually used to generate fat-specific KO models, works in both WAT and BAT [138]. Hence, the development of a technical method that allows conditional gene deletion to specifically target WAT is desired.

Regulation of mitochondrial dynamics is attributed to not only mitochondrial biogenesis, which is enhanced by PGC-1α, but also mitochondrial degradation by autophagic clearance, namely “mitophagy” [139]. Mitophagy is implicated in alterations in characteristics of adipocytes, especially beiging [139]. Therefore, a balance of mitochondrial biogenesis and mitophagy is probably important for maintenance of mitochondrial function in WAT, and regulation of PGC-1α may be involved in this balance.

**Figure 1:**
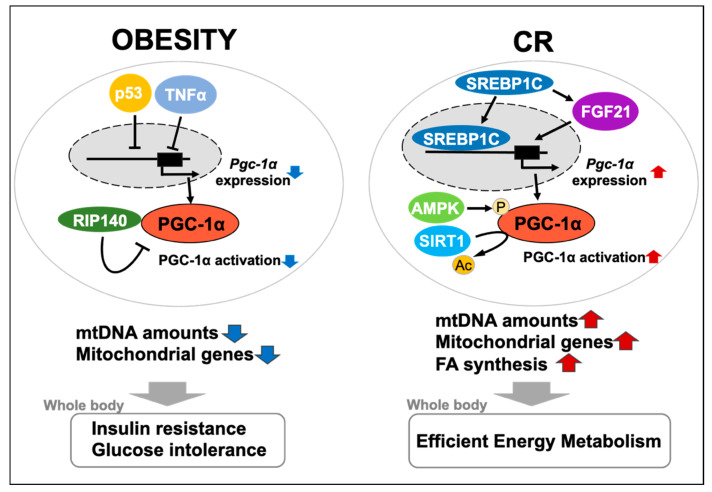
Regulation of PGC-1α in white adipose tissue (WAT) and its impact on whole-body metabolism. In obese WAT, peroxisome proliferator-activated receptor γ coactivator-1α (PGC-1α) is transcriptionally silenced by p53 and tumor necrosis factor α (TNFα), and suppressed at the activity level by interacting with receptor-interacting protein 140 (RIP140). This triggers a decrease in the amount of mtDNA and expression of mitochondrial genes in WAT, resulting in obesity-related pathology (e.g., insulin resistance and glucose intolerance). In WAT, during caloric restriction (CR), PGC-1α is transcriptionally upregulated by sterol regulatory element-binding protein 1c (SREBP-1c) and via the SREBP-1c-fibroblast growth factor 21 (FGF21) axis, and post-translationally activated by AMP-activated protein kinase (AMPK) and Sirtuin 1 (SIRT1). This induces fatty acid (FA) synthesis, in addition to increasing mtDNA and mitochondrial gene expression in WAT, resulting in efficient energy metabolism in the whole body.

In conclusion, much of the evidence introduced in this review strongly indicates that PGC-1α is a major player in regulating mitochondrial biogenesis or function in WAT in response to a systemic metabolic state (Figure 1). Further investigation of WAT- or white adipocyte-specific function of PGC-1α will lead to identification of the novel mechanisms underlying the relationship between mitochondria and obesity- or CR-related physiological changes.

## Data Availability

Not applicable.
